# Methods of creatine kinase-MB analysis to predict mortality in patients with myocardial infarction treated with reperfusion therapy

**DOI:** 10.1186/1745-6215-14-123

**Published:** 2013-05-02

**Authors:** Renato D Lopes, Yuliya Lokhnygina, Victor Hasselblad, Kristin L Newby, Eric Yow, Christopher B Granger, Paul W Armstrong, Judith S Hochman, James S Mills, Witold Ruzyllo, Kenneth W Mahaffey

**Affiliations:** 1 Duke Clinical Research Institute, Duke University Medical Center, 2400 Pratt Street, Room 0311, Terrace Level, Box 3850, Durham, NC, 27705, USA; 2 University of Alberta, 116 Street, Edmonton, Alberta, T6G 2R3, Canada; 3 New York University School of Medicine, 545 1st Avenue, New York, NY, 10016, USA; 4 Institute of Cardiology, Alpejska 42, Warsaw, 04-628, Poland

**Keywords:** Creatine kinase-MB, Infarct size, ST-segment elevation myocardial infarction, Clinical outcomes

## Abstract

**Background:**

Larger infarct size measured by creatine kinase (CK)-MB release is associated with higher mortality and has been used as an important surrogate endpoint in the evaluation of new treatments for ST-segment elevation myocardial infarction (STEMI). Traditional approaches to quantify infarct size include the observed CK-MB peak and calculated CK-MB area under the curve (AUC). We evaluated alternative approaches to quantifying infarct size using CK-MB values, and the relationship between infarct size and clinical outcomes.

**Methods:**

Of 1,850 STEMI patients treated with reperfusion therapy in the COMplement inhibition in Myocardial infarction treated with Angioplasty (COMMA) (percutaneous coronary intervention (PCI)-treated) and the COMPlement inhibition in myocardial infarction treated with thromboLYtics (COMPLY) (fibrinolytic-treated) trials, 1,718 (92.9%) (COMMA, n = 868; COMPLY, n = 850) had at least five of nine protocol-required CK-MB measures. In addition to traditional methods, curve-fitting techniques were used to determine CK-MB AUC and estimated peak CK-MB. Cox proportional hazards modeling assessed the univariable associations between infarct size and mortality, and the composite of death, heart failure, shock and stroke at 90 days.

**Results:**

In COMPLY, CK-MB measures by all methods were significantly associated with higher mortality (hazard ratio range per 1,000 units increase: 1.09 to 1.13; hazard ratio range per 1 standard deviation increase: 1.41 to 1.62; *P* <0.01 for all analyses). In COMMA, the associations were similar but did not reach statistical significance. For the composite outcome of 90-day death, heart failure, shock and stroke, the associations with all CK-MB measures were statistically significant in both the COMMA and COMPLY trials.

**Conclusions:**

Sophisticated curve modeling is an alternative to infarct-size quantification in STEMI patients, but it provides information similar to that of more traditional methods. Future studies will determine whether the same conclusion applies in circumstances other than STEMI, or to studies with different frequencies and patterns of CK-MB data collection.

## Background

The relationship between creatine kinase (CK)-MB measured in the blood and infarct size has been well-established through both pathological and imaging correlations [1-8]. Furthermore, infarct size, measured by release of CK-MB for example, is associated with short- and long-term cardiovascular complications such as cardiac death, reinfarction, congestive heart failure, stroke and unstable angina requiring hospitalization [9-19]. Infarct size determined by other methods of quantification, such as single-photon emission computed tomography, left ventricular angiography, echocardiogram and contrast-enhanced magnetic resonance imaging, have also been associated with subsequent clinical outcomes in patients with ST-segment elevation myocardial infarction (STEMI) [8,20-22].

Based on these known relationships, CK-MB is an important surrogate endpoint in the evaluation of new treatments for STEMI. However, there has been debate about what is the best method for quantifying CK-MB release over the first 72 hours in patients with STEMI. Thus, the optimal method for quantifying infarct size using CK-MB is unknown, as is the nature of the relationships between infarct size and clinical outcomes according to the method used. Therefore, we examined the relationships between infarct size (quantified by multiple methods) using CK-MB and clinical outcomes in patients with STEMI who were enrolled in two phase II randomized clinical trials.

## Methods

We studied 1,850 patients with STEMI treated with reperfusion therapy who were enrolled in the COMplement inhibition in Myocardial infarction treated with Angioplasty (COMMA) (n = 929; PCI-treated) and COMPlement inhibition in myocardial infarction treated with thromboLYtics (COMPLY) (n = 921; fibrinolytic-treated) trials. These trials’ methods have been previously published [23,24]. In brief, the primary objective of COMMA was to determine whether treatment of acute STEMI within 6 hours of onset of symptoms with 1. a bolus of pexelizumab and reperfusion therapy with percutaneous transluminal coronary angioplasty (PTCA), or 2. a bolus and infusion of pexelizumab and PTCA reperfusion therapy, was superior to PTCA reperfusion therapy alone in decreasing infarct size. The primary objective of COMPLY was to determine whether treatment of acute STEMI within 6 hours of onset of symptoms with 1. a bolus of pexelizumab and thrombolytic reperfusion therapy, or 2. a bolus and infusion of pexelizumab and thrombolytic reperfusion therapy, was superior to fibrinolytic reperfusion therapy alone in decreasing infarct size. In both COMMA and COMPLY, CK-MB values were measured at baseline prior to study drug administration, and then at 1.5, 4, 8, 12, 16, 24, 36, 48 and 72 hours postinfusion of the bolus of study drug. In these studies, all CK-MB measures were performed in a central core laboratory using a mass assay.

The CARDINAL trials complied with the Declaration of Helsinki. All patients enrolled provided written informed consent, and the trial was approved by the institutional review boards and ethics committees of participating sites. Use of trial data for the current analyses was approved by the institutional review board of the Duke University Medical Center under protocol number PRO00005720.

### Statistical methods

After excluding 132 patients who did not have at least five serial CK-MB measures (61 from COMMA and 71 from COMPLY), our final analysis population included 1,718 STEMI patients. We determined the observed CK-MB peak numerical value from among the available CK-MB measures and calculated the observed CK-MB area under the curve (AUC) using the standard trapezoidal method. In addition, we used modeling techniques to estimate the CK-MB AUC and fitted peak CK-MB values, as described in the following section. All individual data points were used to fit the models in our analyses.

#### CK-MB curve modeling

Shell *et al.*[25] first described the use of the log-normal curve to express the pattern of CK-MB values for a given person over time. Vollmer *et al.*[26] compared several different models to describe this same pattern, including one that they indicated was equivalent to the log-normal curve. In fact, that curve was similar but not equivalent. They also included the 2-compartment model, the gamma model and the model of Schwerdt *et al.*[27]. Vollmer *et al.* concluded that their version of the log-normal model provided a better fit than the other models. The general form of the log-normal model is as follows:

(1) $$\begin{array}{l} y=\gamma +\beta \frac{\mathrm{Exp}\left\{-\frac{{\left[\mathrm{Ln}\left(t\right)-\mathrm{Ln}\left(\mu \right)\right]}^{2}}{2\sigma^{2}}\right\}}{\sqrt{2\pi }\mathrm{\sigma t}}\;\mathrm{for}\;t>0;\;y\\ \;=\gamma \;\mathrm{for}\;t\leq 0. \end{array}$$

If there is reason to believe that the infarct does not start at time zero but rather at an onset time *δ* (*δ* can be positive or negative), then the model becomes as follows:

(2) $$\begin{array}{l} y=\gamma +\beta \frac{\mathrm{Exp}\left\{-\frac{{\left[\mathrm{Ln}\left(t-\delta \right)-\mathrm{Ln}\left(\mu \right)\right]}^{2}}{2\sigma^{2}}\right\}}{\sqrt{2\pi }\sigma \left(t-\delta \right)}\;\mathrm{for}\;t>\delta ;\;y\\ \;=\gamma \;\mathrm{for}\;t\leq \delta . \end{array}$$

**Figure 1:**
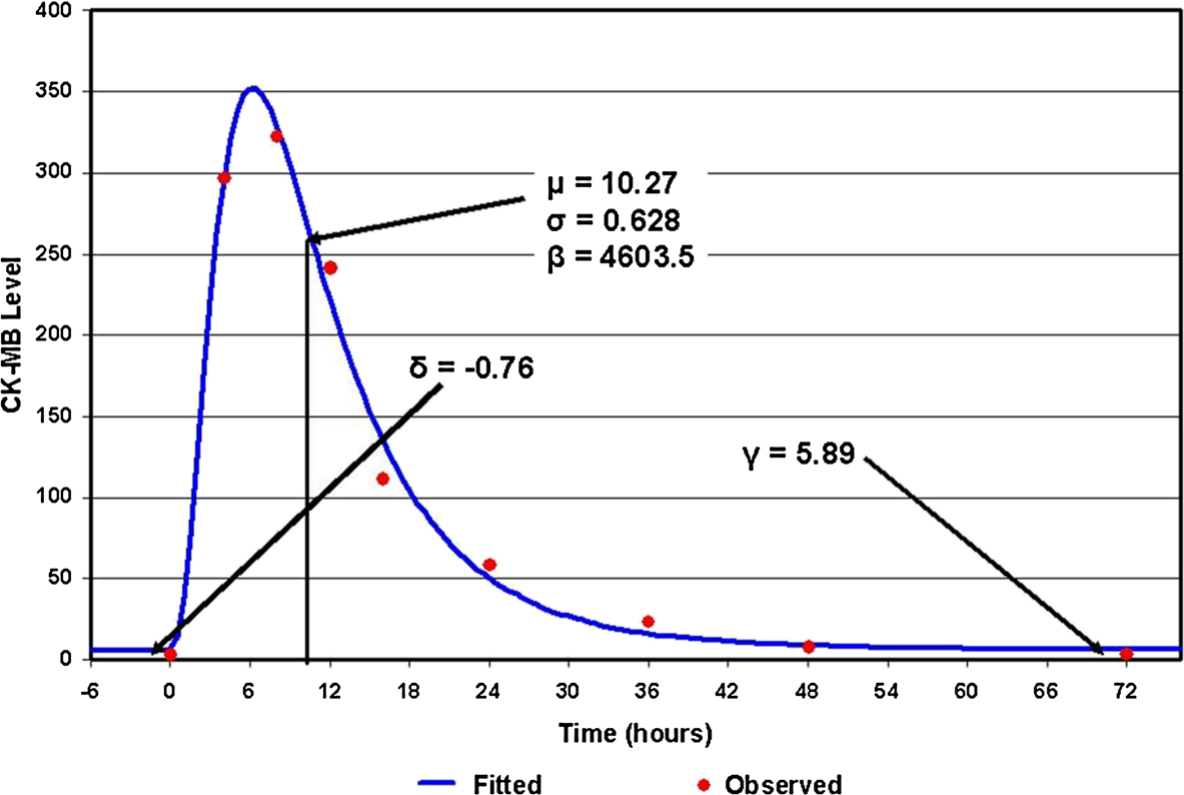
**Example of log-normal cumulative distribution fitted to COMMA.** CK, creatine kinase.

All of the parameters can be estimated using standard maximum likelihood methods, except for *δ*. If we fix *δ*, a very good general method for obtaining least squares estimates of the remaining parameters of a model is the modified Gauss-Newton method, as described by Hartley [28]. The sum of squares of error can be computed for each *δ* and the *δ* producing the minimum error is the optimal estimate. An example of the fit for a given patient in COMMA is shown in Figure 1.

The parameter *δ* can be interpreted as the time to the onset of the event (as measured by the marker). The parameter *γ* is the estimate of the background CK-MB level for a particular individual. The parameter *μ* determines where the curve peaks. Although this parameter is usually the most important when working with log-normally distributed data, it may be the least important in modeling the time course of CK-MB. The parameter *σ* determines how tight (wide) the curve is. If a drug reduces the washout time for CK-MB without affecting the AUC, then this parameter might be a sensitive measure. The parameter *β* determines the total AUC above background (over all time). The area above background from time zero to time *T* is just as follows:

(3) $$\begin{array}{l} \beta \left\{\Phi \left(\left[\mathrm{Ln}\left(T–\delta \right)–\mathrm{Ln}\left(\mu \right)\right]/\sigma \right)-\Phi \left(\left[\mathrm{Ln}(-\delta \right)–\mathrm{Ln}\left(\mu \right)\right]/\sigma )\right\}\;\mathrm{if}\;\delta <0,\\ \beta \left\{\Phi \left(\left[\mathrm{Ln}\left(T–\delta \right)–\mathrm{Ln}\left(\mu \right)\right]/\sigma \right)\right\}\;\mathrm{otherwise}, \end{array}$$

where *Φ*(*x*) is the standard cumulative normal distribution function. If the total area is desired, then *T* × *γ* must be added to the area. In this particular case, *T* = 72 hours.

The fitted peak can be obtained by evaluation of equation (1) or (2) at the timepoint defined by (4):

(4) $$t=\delta +\mu e^{-\sigma^{2}}.$$

#### Analyses of clinical endpoints

We used Cox proportional hazards models to explore the univariable association of CK-MB AUC and peak values derived from both 1. observation and 2. modeling with 90-day mortality and a 90-day composite of death, heart failure, shock and stroke. Pexelizumab had a neutral effect on infarct size in both the COMMA and COMPLY studies; therefore, we did not account for the study drug in our analyses. Statistical significance was defined at the 2-sided *α* = 0.05 level. Data analyses were performed with SAS software (version 8.2; SAS Institute, Cary, NC, USA).

#### Sensitivity analysis

We performed a sensitivity analysis excluding patients with Killip class IV and creatinine clearance (CrCl) <60 ml/min (COMMA, n = 201; COMPLY, n = 200), leaving 616 COMMA patients and 606 COMPLY patients to test whether the relationships between CK-MB measures and clinical outcomes were similar to those in the main analysis.

## Results

### Data quality and model fitting

Table 1 gives the distribution of the number of CK-MB samples per patient. The majority of patients in both studies had between eight and ten CK-MB measures.

**Table 1:** Distribution of samples per patient

**Number of samples**	**Number (%) of patients**	
	**COMMA**	**COMPLY**
<5	61 (6.6)	71 (7.7)
5	17 (1.8)	11 (1.2)
6	49 (5.3)	20 (2.2)
7	113 (12.2)	35 (3.8)
8	233 (25.1)	141 (15.3)
9	453 (49.8)	639 (69.4)
10	3 (0.3)	4 (0.4)

COMMA, COMplement inhibition in Myocardial infarction treated with Angioplasty; COMPLY, COMPlement inhibition in myocardial infarction treated with thromboLYtics.

#### COMMA trial

Of the 61 patients who did not have at least five CK-MB values (6.6% of the total population of patients with CK-MB data), 13 patients died and one patient was discharged during the first 72 hours. Of the 868 patients who had five or more CK-MB levels, 817 were analyzed (exclusions for the COMMA analyses are provided in Table 2).

**Table 2:** Summary of exclusions in the analyses of COMMA patients

	**Placebo**	**Bolus**	**Bolus + infusion**
Patients with available CK-MB values^a^	302	311	316
Excluded patients			
<5 points	18	20	23
No infarct	12	8	5
Could not fit	2	0	0
Delayed infarct	5	2	5
Second infarct	6	3	3
Final sample size for CK-MB analysis	259	278	280

CK, creatine kinase; COMMA, COMplement inhibition in Myocardial infarction treated with Angioplasty. ^a^Patients with at least 5 serial CK-MB measures.

#### COMPLY trial

Of the 71 patients who did not have five readings (7.7% of the total population of patients with CK-MB data), 36 patients died and one patient was discharged during the first 72 hours. Of the 850 patients who had five or more CK-MB levels, 806 were analyzed (exclusions are shown in Table 3).

**Table 3:** Summary of exclusions in the analyses of COMPLY patients

	**Placebo**	**Bolus**	**Bolus + infusion**
Patients with available CK-MB values^a^	307	303	311
Excluded patients			
<5 points	23	23	25
No infarct	6	8	8
Could not fit	0	1	1
Delayed infarct	2	2	2
Second infarct	4	5	5
Final sample size for CK-MB analysis	272	264	270

CK, creatine kinase; COMPLY, COMPlement inhibition in myocardial infarction treated with thromboLYtics. ^a^Patients with at least 5 serial CK-MB measures.

### Clinical outcomes

In the COMPLY trial, there were 49 deaths and 123 composite outcomes. In the COMMA trial, there were only 19 deaths and 72 composite clinical events. In the COMPLY trial, all CK-MB measures were significantly associated with higher rates of 90-day mortality (Table 4). In the COMMA trial, the associations were nearly the same as in the COMPLY trial but failed to reach statistical significance (Table 4). For the composite outcome of 90-day death, heart failure, shock and stroke, the associations with all CK-MB measures were statistically significant in both the COMMA and COMPLY trials (Table 5). For each of these analyses, results were similar when patients with Killip class IV and CrCl <60 ml/min were excluded from the analysis (data not shown). Overall, larger infarct size (per each 1000 units increase in CK-MB) was associated with an approximate 10% increased risk of death and composite of death, heart failure, shock and stroke at 90 days (Tables 4 and 5). To compare how various measures of CK-MB affected 90-day outcomes, we rescaled each measure in units of its standard deviation and evaluated hazard ratios per increase of one standard deviation. As Table 4 shows, the magnitude of association with 90-day outcomes was generally similar between model-derived and observed CK-MB measures. The estimated hazard ratios of 90-day outcomes associated with an increase of one standard deviation in CK-MB AUC (whether observed or model-derived) were larger than hazard ratios associated with increase in peak CK-MB (whether observed or model-derived). However, this finding was not statistically significant.

**Table 4:** Univariable associations of infarct size determined by various methods with 90-day mortality

**Method**	**COMMA trial**		**COMPLY trial**	
	**Hazard ratio (95% CI)**	**Wald *χ***^**2 **^**(*P *** **value)**	**Hazard ratio (95% CI)**	**Wald *χ*** **2 (*P *** **value)**
Total CK-MB AUC, 0 to 72 hours from randomization estimated by curve modeling^a^				
per 1000 units	1.07	2.76	1.09	26.05
	(0.99 to 1.17)	(0.10)	(1.06 to 1.13)	(<0.01)
per 1 SD	1.35		1.58	
	(0.95 to 1.92)		(1.33 to 1.88)	
Estimated peak^a^				
per 100 units	1.02	0.76	1.12	16.41
	(0.98 to 1.07)	(0.38)	(1.06 to 1.19)	(<0.01)
per 1 SD	1.10		1.41	
	(0.89 to 1.36)		(1.20 to 1.67)	
Observed trapezoidal CK-MB AUC				
per 1000 units	1.09	4.61	1.10	29.04
	(1.01 to 1.19)	(0.03)	(1.06 to 1.14)	(<0.01)
per 1 SD	1.45		1.62	
	(1.03 to 2.03)		(1.36 to 1.93)	
Observed peak				
per 100 units	1.16	3.75	1.13	16.26
	(1.00 to 1.34)	(0.05)	(1.06 to 1.19)	(<0.01)
per 1 SD	1.41		1.41	
	(1.00 to 2.00)		(1.19 to 1.66)	

^a^Estimated from the log-normal model. AUC, area under the curve; CI, confidence interval; CK, creatine kinase; COMMA, COMplement inhibition in Myocardial infarction treated with Angioplasty; COMPLY, COMPlement inhibition in myocardial infarction treated with thromboLYtics; SD, standard deviation.

**Table 5:** Univariable associations of infarct size determined by various methods with 90-day composite of mortality, heart failure, shock and stroke

**Method**	**COMMA trial**		**COMPLY trial**	
	**Hazard ratio (95% CI)**	**Wald *χ***^**2 **^**(*P *** **value)**	**Hazard ratio (95% CI)**	**Wald *χ***^**2 **^**(*P *** **value)**
Total CK-MB AUC 0 to 72 hours from randomization estimated by curve modeling^a^				
per 1000 units	1.14	53.08	1.08	48.31
	(1.10 to 1.18)	(<0.001)	(1.06 to 1.11)	(<0.001)
per 1 SD	1.74		1.51	
	(1.50 to 2.02)		(1.35 to 1.70)	
Estimated peak^a^				
per 100 units	1.03	15.34	1.11	29.87
	(1.02 to 1.05)	(<0.001)	(1.07 to 1.15)	(<0.001)
per 1 SD	1.15		1.36	
	(1.07 to 1.24)		(1.22 to 1.53)	
Observed trapezoidal CK-MB AUC				
per 1000 units	1.15	55.76	1.09	50.26
	(1.11 to 1.19)	(<0.001)	(1.06 to 1.11)	(<0.001)
per 1 SD	1.79		1.53	
	(1.53 to 2.08)		(1.36 to 1.73)	
Observed peak				
per 100 units	1.26	45.96	1.11	30.05
	(1.18 to 1.34)	(<0.001)	(1.07 to 1.16)	(<0.001)
per 1 SD	1.72		1.36	
	(1.47 to 2.01)		(1.22 to 1.52)	

^a^Estimated from the log-normal model. AUC, area under the curve; CI, confidence interval; CK, creatine kinase; COMMA, COMplement inhibition in Myocardial infarction treated with Angioplasty; COMPLY, COMPlement inhibition in myocardial infarction treated with thromboLYtics; SD, standard deviation.

In COMPLY, all four measures of infarct size were highly correlated with each other (correlation ≥0.93). In COMMA, observed AUC and peak and estimated AUC were highly correlated (correlation ≥0.96); however, the correlations between estimated peak and three other measures were much lower (correlations 0.51 to 0.56).

## Discussion

Biomarkers such as CK-MB are used for diagnosis of myocardial infarction (MI), but they can also be used to estimate infarct size as a surrogate endpoint in clinical trials of new treatments for MI [6,29-33]. Many methods are used to determine infarct size, but little comparative evidence exists as to which method best describes the relationship of infarct size with meaningful clinical outcomes, including mortality. Our results show that, in general, the association of larger infarct size and worse clinical outcomes, including mortality, is significant and similar using multiple observed and derived measures of infarct size based on CK-MB.

The use of infarct size as a surrogate outcome in phase II clinical trials as a preliminary estimate of treatment effect is predicated on the previously demonstrated relationship between CK-MB and infarct size [4,5,7,8], and between infarct size and meaningful clinical outcomes [8-22]. Thus, it is important to define the most accurate and reliable method to estimate infarct size using CK-MB measures as surrogate endpoints in clinical trials of treatment effect. Our main goal in the present study was to illustrate methodological variations in the use of CK-MB to estimate infarct size, and to explore the relationship between infarct size (determined by these various methods) and clinical outcomes. We believe the insights from our work will be of particular relevance for phase I and II studies. In addition, we believe the questions we addressed and our results may be applicable to troponin measures as well.

Estimating infarct size based on measurements of CK-MB in blood has inherent challenges. First, the fact that pathological findings are the confirmatory and gold standard criteria for MI size is a limitation *per se*; however, using enzymatic measurement in this setting may underestimate infarct size from 14% to 47% of the time, especially if not all CK-MB values are available [34]. Despite this, a previous study in patients who did not receive reperfusion therapy reported good correlation between the enzymatic estimates of infarct size based on CK-MB and pathological measurements [35]. Most importantly, the time-activity curve of CK-MB in the bloodstream is influenced by reperfusion, and because reperfusion itself influences infarct size, it may complicate the estimation of infarct size by CK-MB analysis [5,7,33]. In our study, all patients underwent reperfusion. Thus, any possible change in the CK-MB-release ratio between reperfused and not reperfused patients or challenges in interpreting the results based on such heterogeneity was avoided. One might have expected that there would be more heterogeneity in reperfusion speed or completeness among fibrinolytic-treated patients in COMPLY than in PCI-treated patients in COMMA, and thus, a less-tight relationship with outcomes. However, the opposite was observed. Because there were many more events in COMPLY than in COMMA, while the effect sizes were comparable, we believe the most likely explanation for the lack of significance in COMMA was one of statistical power.

Some believe that using cumulative CK-MB value is more accurate to estimate MI size; however, cumulative value requires serial acquisition of blood samples, which is both costly and time-consuming.

We found that the log-normal model we used to estimate AUC and peak CK-MB did an outstanding job of describing the time course of CK-MB readings in patients having a single infarct. We also showed that the observed CK-MB measures (AUC and peak) and measures obtained from sophisticated curve fitting had comparable associations with endpoints of 90-day death and 90-day death, heart failure, shock or stroke. However, this may not be true in studies in which CK-MB values are measured less often. At least five CK-MB measurements are necessary to fit a log-normal model for the CK-MB curve. It is still possible to calculate observed CK-MB AUC using fewer than five measurements, but the validity is questionable. Given the similarity in relationships with clinical outcomes, our results suggest that the method selected for quantifying infarct size as a surrogate endpoint in clinical trials of STEMI treatment can vary without compromising validity or generalizability of the trial results, depending on the following: the operational needs and capacity of the trial and its sites, available funding, and the quantitative skills and expertise of the trial team.

### Strengths and limitations

COMMA and COMPLY patients were STEMI patients enrolled shortly after onset of MI symptoms, and the majority had at least eight CK-MB samples collected within 72 hours of enrollment. Thus, collecting CK-MB readings over time and using them as a primary endpoint measure in early-phase clinical trials is clearly feasible. Further, the dataset itself was of extremely high quality. More than 7200 CK-MB measurements were taken from more than 900 patients and the number of questionable values was extremely small. However, it is unknown whether our conclusions apply to studies with different frequencies and patterns of CK-MB data collection or to non-STEMI populations. We did not collect information about skeletal muscle disease, but we believe such cases would have been rare in these trials and would not have substantially influenced our results. Finally, although we believe that the methods and issues that we described and analyzed in the current study may be applied for troponin as well, we did not have troponin data (or available samples) to confirm this hypothesis.

## Conclusions

Sophisticated curve modeling of serial CK-MB measures in STEMI patients provides an alternative method of quantifying infarct size, and it produces results that are comparable to more standard methods used in determining observed peak or AUC of CK-MB; however, it requires intensive serial sampling regimens that may not be feasible in all trials.

## Abbreviations

AUC: Area under the curve; CK: Creatine kinase; COMMA: COMplement inhibition in Myocardial infarction treated with Angioplasty; COMPLY: COMPlement inhibition in myocardial infarction treated with thromboLYtics; CrCl: Creatinine clearance; MI: Myocardial infarction; PCI: Percutaneous coronary intervention; PTCA: Percutaneous transluminal coronary angioplasty; STEMI: ST-segment elevation myocardial infarction.

## Competing interests

Paul W Armstrong declares research support from Procter & Gamble Pharmaceuticals (Mason, OH, USA) and Alexion Pharmaceuticals (Cheshire, CT, USA). Witold Ruzyllo declares research support from Procter & Gamble Pharmaceuticals and Alexion Pharmaceuticals. The remaining authors declare that they have no competing interests.

## Authors’ contributions

RDL contributed to the study concept and design; acquisition, analysis and interpretation of data; drafting and critical revision of the manuscript for important intellectual content; and acquisition of funding. YL contributed to the study concept and design; acquisition, analysis and interpretation of the data; critical revision of the manuscript for important intellectual content; and statistical analysis. VH contributed to the study concept and design; acquisition, analysis and interpretation of the data; critical revision of the manuscript for important intellectual content; and statistical analysis. LKN contributed to the acquisition, analysis and interpretation of the data as well as critical revision of the manuscript for important intellectual content. EY contributed to the acquisition, analysis and interpretation of the data; critical revision of the manuscript for important intellectual content; and statistical analysis. CBG and PWA contributed to the critical revision of the manuscript for important intellectual content and acquisition of funding. JSH, JSM, and WR contributed to the critical revision of the manuscript for important intellectual content. KWM contributed to the study concept and design; analysis and interpretation of the data; critical revision of the manuscript for important intellectual content; and acquisition of funding. All authors read and approved the final manuscript.
